# Editorial: Reviews in molecular and cellular oncology

**DOI:** 10.3389/fonc.2023.1224902

**Published:** 2023-06-08

**Authors:** Daniel P. Bezerra, Jie Ni, Maoshan Chen

**Affiliations:** ^1^ Gonçalo Moniz Institute, Oswaldo Cruz Foundation (IGM-FIOCRUZ/BA), Salvador, Bahia, Brazil; ^2^ Cancer Care Centre, St George Hospital, Kogarah, NSW, Australia; ^3^ St George and Sutherland Clinical School, Faculty of Medicine, UNSW, Sydney, NSW, Australia; ^4^ Laboratory of Radiation Biology, Department of Blood Transfusion, Laboratory Medicine Centre, The Second Affiliated Hospital, Army Medical University, Chongqing, China

**Keywords:** cellular oncology, hallmarks of cancer, reviews - articles, targeted therapy, molecular oncology studies

Cancer is an important public health problem worldwide. According to the latest global cancer burden update article using GLOBOCAN 2020, 19.3 million new cancer cases (18.1 million excluding nonmelanoma skin cancer) and nearly 10.0 million cancer deaths (9.9 million excluding nonmelanoma skin cancer) occurred worldwide in 2020 (1). On the other hand, since 1991, the cancer death rate has continuously decreased in some regions of the world, resulting in a 33% overall decrease and an estimated 3.8 million cancer deaths avoided in the United States of America (2). In particular, part of these improvements in survival is due to better knowledge of the molecular and cellular processes that drive cancer progression and metastasis, which has been fundamental in the development of targeted therapies for the treatment of cancer.

The current hallmarks of cancer encompass various characteristics such as evading growth suppressors; avoiding immune destruction; activating invasion and metastasis; senescent cells; genome instability and mutation; resisting cell death; and sustaining proliferative signaling (3). A better understanding of these cancer hallmarks and the molecular mechanisms that are responsible for these processes, will enable the development of effective and novel therapeutic modalities and improve the quality of life and survival of cancer patients. This Research Topic aimed to highlight recent advances in the field while emphasizing important directions and new possibilities for future inquiries.

In particular, Wang et al. reviewed the role of adipokines in pancreatic cancer. Adipokines are cytokines, such as leptin, adiponectin, and oncostatin-M, produced by adipose tissue that play functional roles in obesity, inflammation, the body’s energy/metabolic state, etc. (4). Leptin signaling increases MMP-13 synthesis, which promotes cell invasion and metastasis in human pancreatic cancer (5). Leptin also increases pancreatic tumor cell motility by activating the PI3K/AKT pathway (6). Likewise, adiponectin inhibits the growth of human pancreatic cancer by inhibiting the β-catenin signaling pathway (7), and oncostatin-M induces potent epithelial-mesenchymal transition and cancer stem cell phenotypes in pancreatic cancer (8).

In another article of this Research Topic, Zhou et al. reviewed patient-derived organoids (PDOs) in precision medicine. PDOs is a tool for individualized medical decisions that predicts patients’ reactions to therapy regimens and may enhance treatment results. Interestingly, Wnt-activated PDOs of Barrett’s esophagus exhibited histologic atypia, increased proliferative and replicative activity, decreased apoptosis, and longer cultivability (9).

In conclusion, molecular oncology represents a paradigm shift in cancer research, bringing together multidisciplinary efforts and cutting-edge technologies to tackle cancer at its core. This Research Topic provide a new update in this field that will direct further research to improve cancer prevention and treatment.

## Author contributions

DB prepared the first version of the editorial. DB, JN and MC - discussed the editorial content and revised the final editorial text. All authors contributed to the article and approved the submitted version.

## References

[1] Sung H Ferlay J Siegel RL Laversanne M Soerjomataram I Jemal A. Global cancer statistics 2020: GLOBOCAN estimates of incidence and mortality worldwide for 36 cancers in 185 countries. CA Cancer J Clin (2021) 71(3):209–49. doi: 10.3322/caac.21660 33538338

[2] Siegel RL Miller KD Wagle NS Jemal A. Cancer statistics, 2023. CA Cancer J Clin (2023) 73(1):17–48. doi: 10.3322/caac.21763 36633525

[3] Hanahan D. Hallmarks of cancer: new dimensions. Cancer Discovery (2022) 12(1):31–46. doi: 10.1158/2159-8290.CD-21-1059 35022204

[4] Ouchi N Parker JL Lugus JJ Walsh K. Adipokines in inflammation and metabolic disease. Nat Rev Immunol (2011) 11(2):85–97. doi: 10.1038/nri2921 21252989 PMC3518031

[5] Fan Y Gan Y Shen Y Cai X Song Y Zhao F. Leptin signaling enhances cell invasion and promotes the metastasis of human pancreatic cancer *via* increasing MMP-13 production. Oncotarget (2015) 6(18):16120–34. doi: 10.18632/oncotarget.3878 PMC4599260 25948792

[6] Mendonsa AM Chalfant MC Gorden LD VanSaun MN. Modulation of the leptin receptor mediates tumor growth and migration of pancreatic cancer cells. PloS One (2015) 10(4):e0126686. doi: 10.1371/journal.pone.0126686 25919692 PMC4412670

[7] Jiang J Fan Y Zhang W Shen Y Liu T Yao M. Adiponectin suppresses human pancreatic cancer growth through attenuating the β-catenin signaling pathway. Int J Biol Sci (2019) 15(2):253–64. doi: 10.7150/ijbs.27420 PMC6367542 30745818

[8] Smigiel JM Parameswaran N Jackson MW. Potent EMT and CSC phenotypes are induced by oncostatin-m in pancreatic cancer. Mol Cancer Res (2017) 15(4):478–88. doi: 10.1158/1541-7786.MCR-16-0337 PMC5380554 28053127

[9] Liu X Cheng Y Abraham JM Wang Z Wang Z Ke X. Modeling wnt signaling by CRISPR-Cas9 genome editing recapitulates neoplasia in human Barrett epithelial organoids. Cancer Lett (2018) 436:109–18. doi: 10.1016/j.canlet.2018.08.017 PMC6152930 30144514

